# Multi-Complementary Model for Long-Term Tracking

**DOI:** 10.3390/s18020527

**Published:** 2018-02-09

**Authors:** Deng Zhang, Junchang Zhang, Chenyang Xia

**Affiliations:** School of Electronics and Information, Northwestern Polytechnical University, Xi’an 710072, China; zhangjc@nwpu.edu.cn (J.Z.); cyxia2016@mail.nwpu.edu.cn (C.X.)

**Keywords:** multi-complementary model, object detection map, detection module, scale calculation optimization

## Abstract

In recent years, video target tracking algorithms have been widely used. However, many tracking algorithms do not achieve satisfactory performance, especially when dealing with problems such as object occlusions, background clutters, motion blur, low illumination color images, and sudden illumination changes in real scenes. In this paper, we incorporate an object model based on contour information into a Staple tracker that combines the correlation filter model and color model to greatly improve the tracking robustness. Since each model is responsible for tracking specific features, the three complementary models combine for more robust tracking. In addition, we propose an efficient object detection model with contour and color histogram features, which has good detection performance and better detection efficiency compared to the traditional target detection algorithm. Finally, we optimize the traditional scale calculation, which greatly improves the tracking execution speed. We evaluate our tracker on the Object Tracking Benchmarks 2013 (OTB-13) and Object Tracking Benchmarks 2015 (OTB-15) benchmark datasets. With the OTB-13 benchmark datasets, our algorithm is improved by 4.8%, 9.6%, and 10.9% on the success plots of OPE, TRE and SRE, respectively, in contrast to another classic LCT (Long-term Correlation Tracking) algorithm. On the OTB-15 benchmark datasets, when compared with the LCT algorithm, our algorithm achieves 10.4%, 12.5%, and 16.1% improvement on the success plots of OPE, TRE, and SRE, respectively. At the same time, it needs to be emphasized that, due to the high computational efficiency of the color model and the object detection model using efficient data structures, and the speed advantage of the correlation filters, our tracking algorithm could still achieve good tracking speed.

## 1. Introduction

Video tracking is an important part of computer vision and is widely used across a variety of fields including intelligent transportation, man-machine interaction, and military guidance [1,2]. This paper focuses on how to quickly and effectively address the tracking drift problem in object tracking processes when confronted with similarly colored backgrounds, object occlusions, low illumination color images, and sudden illumination changes.

In recent years, correlation filters have attracted more attention for their advantages in efficiency and robustness. In [3], a video tracking algorithm was proposed based on the sum of the mean square error of the minimum output of a correlation filter. Subsequently, Henriques et al. [4] proposed a tracking algorithm based on the circulant structure of tracking-by-detection with kernels (CSK) that used cyclic structure coding to densely sample and train the Regularized Least Squares (RLS) of a nonlinear classifier. Later, CSK was improved with a Kernel Correlation Filter (KCF [5]) that used a Histogram of Oriented Gradients (HOG [6]) features tracking algorithm. Danelljan et al. [7] introduced space regularization in the filter learning and penalized the filter coefficients according to their spatial position. Danelljan et al. [8] used the multi-channel color features to extend CSK and obtained a good performance. Sui et al. [9] greatly improved the filter tracking performance by introducing three sparse correlation-related loss functions into the training of the filter. Convolutional neural network (CNN) features have also demonstrated outstanding results in object classification, image identification, and so on [10,11,12,13,14]. Ma et al. [12] exploited the complementary nature of features extracted from three layers of CNN and used the coarse-to-fine translation estimation for object tracking. Danelljan et al. [14] went beyond the conventional correlation filter framework and learned the correlation filter in the continuous spatial domain of various features, which achieved good tracking performance. However, their algorithm greatly reduced the tracking speed of the correlation filter. In terms of scale calculation, two consistent and relatively independent correlation filters were designed in [15], which achieved object position tracking and scale conversion, respectively. Huang et al. [16,17] integrated the class-agnostic detection proposal method [18] into the correlation filters tracking framework to solve the target scale and aspect ratio of the target deformation problem.

For different models of fusion and different fusion methods, however, tracking performance has not been so good. Kwon et al. [19,20] used complementary trackers to combine different observation and motion models, then integrated their estimation results into a sampling framework. Based on a historical frame and Multiple Experts using Entropy Minimization (MEEM) [21], different Support Vector Machine (SVM) classifiers selected the classifier with the strongest object recognition capacity to decide the tracking result. Using global search, Smith et al. [22] located potential candidate samples marked by contour features, having first obtained better positive and negative samples using weaker classifiers. Classifier tests and updates were then undertaken using stronger detectors. This helped to reduce the classifier search space and false object interference. Similar to the Correlation Filter Based Tracking Algorithm, the color-based tracking algorithm has achieved good performance in terms of speed and performance [23,24]. In the Sum of Template and Pixel-wise Leaners tracker (Staple [25]), Bertinetto et al. took advantage of the complementary sample information by fusing the predictions of the filter model and the color model, therefore showing good performance in handling deformation and fast motion. However, it drifted when target objects underwent heavy illumination variation, occlusion, and background clutters. To further enhance the Staple tracker’s robustness, we not only studied a correlation filter model and a color model, but also integrated an object model based on contours features, which was proposed in Edge Boxes [18], into tracker to generate a multi-complementary model for more robust tracking. Among them, the filter model relies on the spatial distribution of the target object and is relatively sensitive to the deformation. However, the color regression model and the contour-based detection model have good global characteristics and good adaptability to the target deformation. The color model is sensitive to the illumination transformation, but the filter regression and contour-based detection models are adaptable to the illumination changes of the target. The detection model relies only on the edge information of the image and the current size of the online learning ability is poor, but the good online learning ability of the color regression model and the filter regression model are a good supplement to the detection model, and less learning information also reduces the likelihood of elegant filter and color models. Since each model is responsible for the tracking of specific features, the three complementary models are then combined to form a more robust tracking algorithm. At the same time, using efficient data structures, the scores of tens of thousands of candidate boxes can be evaluated in a thousandth of a second [18], and the object detection response scores can be efficiently calculated. 

Currently, tracking detection frameworks are also very popular, playing a key role among numerous recent tracking methods [26,27,28]. To mitigate the stability-plasticity dilemma of online model updating for visual tracking, Kalal et al. [26] decomposed the tracking tasks into Tracking, Learning and Detecting (TLD), where tracking and detecting were mutually promoted. The tracking results provided training data to update the detector, and the detector re-initialized the tracker whenever it failed. This mechanism works well for long-term tracking [26,29,30]. In [31], a long-term filter was proposed that used stochastic sampling to solve the model drifting problem. Predicting objects in combination with multiple estimations can effectively complement each of the tracking methods to deliver a more robust performance. Long-term correlation tracking (LCT) [30] is a classic long-time tracking algorithm, which solves the problems of target deformation, abrupt motion, and heavy occlusion that appear in long-time tracking. In the LCT algorithm, in addition to training the translation filter and the scale filter, a confidence detection filter is also trained from the reliable tracking results. The confidence detection filter can be a good measure of the confidence of the tracking results of the tracking module and the detection results in the detector module. Our algorithm is similar to the LCT algorithm, but we did not retrain an independent confidence filter same as the LCT algorithm. We improved the filter in the tracking module by high confidence updating to obtain our confidence detection filter. Furthermore, we also used the Average Peak-to Correlation Energy (APCE) [32] to determine if the tracking results were reliable at the same time. To improve the robustness of the tracking, the LCT algorithm re-detected the target by training an online random fern classifier after the target tracking failed. In this paper, by combining the object detection model with the edges model and the color model-based histogram feature, we proposed a new detection method algorithm, which not only had good detection accuracy, but also had better detection efficiency than the traditional classifier-based detection method.

Based on the above issues, this paper proposed MMLT (Multi-Complementary model for Long-term Tracking), a long-term object tracker that combines a multiple complementary model. The main goal was to solve the difficulties in real scenes such as object occlusions, background clutters, motion blur, low illumination color images, and sudden illumination changes. The main contributions of our work are as follows.

1. By incorporating the object response model into the Staple algorithm, which combines a correlation filter and color model, the tracking robustness was greatly improved. Each model is responsible for the tracking of specific features and then combined three complementary models for robust visual tracking.
2. Unlike traditional classifier-based object detection, an efficient object detection model with contour features and color histogram features is proposed for the first time, which significantly improved detection efficiency and detection speed.
3. The redundancy aspect of the calculation of image features within each scale of the correlation filter module is optimized in this paper to improve the execution speed of the algorithm.

## 2. Multi-Complementary Model Tracking

Our baseline was the Staple (Sum of Template and Pixel-wise Leaners) algorithm. The Staple algorithm divides the tracking into the translational tracking phase and scale tracking phase. The translation tracking gives the position estimation and the scale tracking phase computes the target scale using a 1D correlation filter. During the translational tracking phase, Staple incorporates the response scores of the color model and correlation filter model (using HOG features) to achieve a good tracking performance. However, as the color information is easily disturbed by factors such as the environment and light, the performance of the tracker is limited. Thus, it is desirable that other additional features should be used as a complement to the color feature to improve the performance of the tracker. In this paper, the proposed MMT (Multi-Complementary Model Tracking), which incorporates the object model based on Edge Boxes [18] in the translational tracking phase of Staple. Edge Boxes [18] is based on the characteristics of the object contour edges information and has good adaptability to light and background changes. By fusing the multi-channel complementary feature response scores, the diversity of the sample information and discriminant can be utilized to a greater extent. This improves the generalizability of the tracker. In addition, we optimize the method of scales calculation, greatly reducing redundant operations and increasing the tracking speed.

Staple adopts the tracking-by-detection paradigm. As the location estimation and scale estimation are separate, they are responsible for their own work. In frame *t* + 1, translation tracking obtains the new target position based on the fix size of target size $s_{t}$ of the previous frame, then scale tracking updates the new scale with the new position computed by translation tracking. Therefore, in translation tracking of frame *t* + 1, given a search patch $d_{t+1}$ extracted around the previous target position, and the fix target size $s_{t}$, the Staple chooses the target bounding box $p_{t+1}$ that gives the target location from a set $ϒ=\left\{\Pi :\;l_{i,j}\left(\Pi \right)\in r,\;\vartheta \left(\Pi \right)=s_{t}\right\}$ to maximize fusing scores:

(1) $$p_{t+1}={\arg\max}_{\Pi \in ϒ}\;\gamma_{M_{0}}f_{0}\left(T_{0}\left(d_{t+1},\Pi \right);M_{0,t}\right)+\gamma_{M_{1}}f_{1}\left(T_{1}\left(d_{t+1},\Pi \right);M_{1,t}\right)$$

where r is a valid inner region; $r\subset p_{t+1}$, $l_{i,j}\left(\Pi \right)\in r$ represents each bounding box Π‘s location (i,j) in the region r; and $\vartheta \left(\Pi \right)=s_{t}$ represents the size of the Π is equal to the fix size $s_{t}$. The functions $T_{0}$ and $T_{1}$ are the image transformation such that $f_{0}\left(T_{0}\left(p_{t+1},\Pi \right);M_{0,t}\right)$ and $f_{1}\left(T_{1}\left(p_{t+1},\Pi \right);M_{1,t}\right)$ assign scores to the bounding box Π according to the color model parameters and $M_{0,t}$ filter model parameters $M_{1,t}$, respectively. In addition, color model parameters $M_{0,t}$ and filter model parameters $M_{1,t}$ are all trained from the previous target state and images, and parameters $\gamma_{M_{0}}$ and $\gamma_{M_{1}}$ represent the combination coefficients of the color model and filter model, respectively.

In this paper, by incorporating the object model to the Staple algorithm, we obtain the target bounding box $p_{t+1}$ to maximize a new fusing score of the three complementary models. The scores function can be represented by:

(2) $$p_{t+1}={\arg\max}_{\Pi \in ϒ}\;\sum_{i=1}^{N_{m}}\gamma_{M_{i}}f_{i}\left(T_{i}\left(d_{t+1},\Pi \right);M_{i,t}\right)\;\;$$

where $N_{m}$, which is three in this paper, is the number of models; the function $T_{i}$ is an image transformation such that $f_{i}\left(T_{i}\left(d_{t+1},\Pi \right);M_{i,t}\right)$ assigns a score to the bounding box Π according to the models parameters $M_{i,t}$ trained from the previous target state and images; and $\gamma_{M_{0}}$, $\gamma_{M_{1}}$ and $\gamma_{M_{2}}$ are the combination coefficients of the color model, filter model, and object model scores, respectively. Moreover, they are renamed $\tau_{c}$, $\tau_{f}$, and $\tau_{o}$, respectively, in this paper.

**Figure 1:**
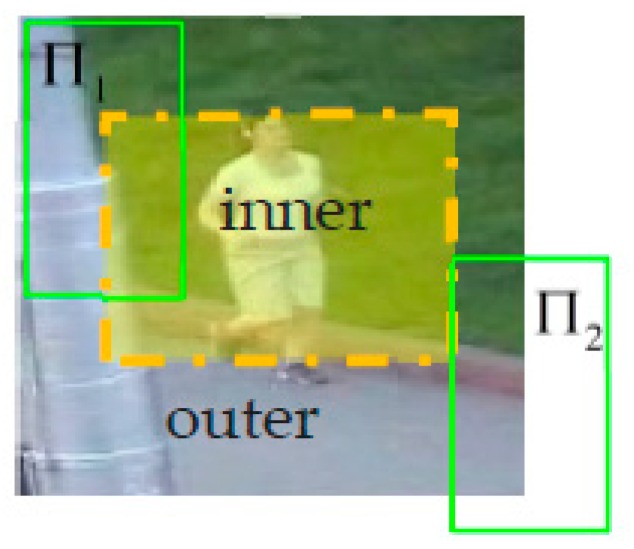
Inner region and outer region of search patch. The region inside the yellow box is the inner region. The region outside of the yellow box and inside the search patch is the outer region.

Before we introduce specific models, we first introduce the detailed concept of the inner region, which will be used in the three models based on a tracking-by-detection principle. Given a search area of $d_{t+1}$ for tracking and a bounding box with a fixed size for the sliding window-based detection method, all the bounding boxes that center at different positions in a detection area of $d_{t+1}$ cannot be used to detect. As shown in Figure 1, the bounding box $\Pi_{2}$ centered the position so that it exceeded the yellow area, which has many pixels out of the overall detection area $d_{t+1}$, so the bounding boxes will not be detected. We named the position set consisting of all the center positions of these bounding box as the outer region, and the region at which the bounding box center can be detected was named as the inner region in this paper. This is labeled with yellow in Figure 1. In addition, the set $ϒ=\left\{\Pi :\;l_{i,j}\left(\Pi \right)\;\in \;r,\;\vartheta \left(\Pi \right)=s_{t}\right\}$ was represented in all boxes (with the fix size of $s_{t}$) centered at different positions in the region r, which was all of the search sample space. Then, we use Multi-Complementary model to calculate the combination scores of all the bounding boxes in ϒ and then estimate the target location at the max value of the combination scores.

Below, we introduce the filter model and color model used in Staple, as well as the object model that we have incorporated in Staple. In Section 2.1 and Section 2.2, we briefly introduce the filter model and color model used in Staple, and, in Section 2.3, we introduce the object detection model based on Edge Boxes [18] that we incorporated in Staple. In Section 2.4, we describe the method of fusing the multiple model predictive response scores, which was used in Staple. Section 2.5 presents how we optimized the scales calculation to significantly improve the speed of the algorithm.

### 2.1. Learning of Filter Model 

In Staple, the filter model is a type of tracking-by-detection model. During the training process, $T_{d,t}$ is a rectangular patch which is sampled from (*t*)-th frame, and the corresponding l dimension Histogram of Oriented Gradients (HOG) feature map $f^{l}$, l∈(1, ⋯,d) is extracted from $T_{d,t}$. Then, by minimizing the objective function, a set of filters h for d dimensional features are trained. The loss function is then:

(3) $$\epsilon ={\left\|g-\overset{d}{\underset{l=1}{\sum }}h^{l}*f^{l}\right\|}^{2}+\lambda \overset{d}{\underset{l=1}{\sum }}{\left\|h^{l}\right\|}^{2}$$

where * represents the circular correlation; $h^{l}$ is the corresponding feature filter for each l dimension; and g is the desired correlation output, which generally selects the Gaussian function with a maximum value of 1. The second parameter λ≥0 represents the coefficient of the regularization term. We then use Parseval’s Theorem for the frequency domain to obtain a fast solution, thus obtaining:

(4) $$\begin{array}{l} H^{l}=\frac{N_{t}^{l}}{D_{t}},\\ N_{t}^{l}=\bar{G}F^{l},\;D_{t}=\overset{d}{\underset{k=1}{\sum }}\;\bar{F^{k}}F^{k}+\lambda . \end{array}l=1,\ldots ,d$$

where $\bar{G}$ is the DFT conjugation of the Gaussian response g; and $F^{k}$ and $\bar{F^{k}}$ are the dot-product operations of the frequency domain of the k-dimensional features map corresponding to the image patch $T_{d,t}$ and the corresponding conjugate operation, respectively.

Based on the above model, we rename the filter $H^{l},\left(l=1,\ldots ,d\right)$ used in the translation phase to the translation filter $R_{c}$, with the corresponding numerator A and denominator B, respectively.

$R_{c}$ is updated with a learning factor $\eta_{f}$:

(5) $$\begin{array}{l} A_{t}^{l}=\left(1-\eta_{f}\right)A_{t-1}^{l}+\eta_{f}\bar{G}F_{t}^{l}\\ B_{t}=\left(1-\eta_{f}\right)B_{t-1}+\eta_{f}\overset{d}{\underset{k=1}{\sum }}\bar{F_{t}^{k}}F_{t}^{k} \end{array}$$

where t is the index of the frame. 

#### Calculating the Filter Scores

Given a search patch $d_{t+1}$, which has a size of $\hat{c}\times \hat{l}$ and its inner region r in (*t+1*)-th frame, the HOG feature map of $d_{t+1}$ is extracted. When the *l*-th dimension of the rectangular features map is marked as $z_{t+1}^{l}$, and its frequency domain is $Z_{t+1}^{l}$, the correlation scores $S_{\left(f,t+1\right)}$ of search area $d_{t+1}$ is obtained by convolving features map $z_{t+1}$ and correlation filter $R_{C}$, which is obtained in the previous frame with Equation (5). The specific formula is as follows:

(6) $$S_{\left(f,t+1\right)}={ℱ}^{-1}\left(\frac{\sum_{l=1}^{d}\bar{A_{t}^{l}}\;Z_{t+1}^{l}}{B_{t}+\lambda }\right)$$

where $A_{t}^{l}$ and $B_{t}$ are the numerator and denominator of the translation filter $R_{c}$ obtained in the previous frame, respectively. ${ℱ}^{-1}$ represents the inverse Discrete Fourier Transform (DFT) operator. 

As the filter model is based on the tracking-by-detection principle, the scores value of a position in the response map can represent the score of the bounding box centered at different positions in search patch $d_{t+1}$ to the object. Unlike the sliding-window-based detection of color and object model, the filter model uses the same properties as the circular convolution, forming the response shares the same size with the feature template (with the same size of $d_{t+1}$). In Figure 1, we show that the inner region r is in the area of search patch $d_{t+1}$, therefore the filter response $y_{t+1}^{f}$ of the inner region r can be obtained by cropping the filter response $S_{\left(f,t+1\right)}$ of a search patch $d_{t+1}$, which corresponds to the scores of all the bounding box in set $ϒ=\left\{\Pi :\;l_{i,j}\left(\Pi \right)\in r,\;\vartheta \left(\Pi \right)=s_{t}\right\}$.

### 2.2. Learning of Color Model

The color model is also based on the widely used tracking-by-detection principle, which selects the bounding box (gives the target position) with the highest score from the bounding box set as the final test result to localize the object of interest within a new frame. As with other classifier-based approaches, color models obtain parameters by learning both the positive and negative samples simultaneously.

We follow Staple, where the color features are based on RGB colors, and the bins color histograms are computed in a 32×32×32 bins space. To have the sparse features to speed up the calculation, in the color model, the Staple algorithm maps each pixel u represented by the RGB space into an index feature j=ϕ(u) in a 32 × 32 × 32 bins space in the image.

**Figure 2:**
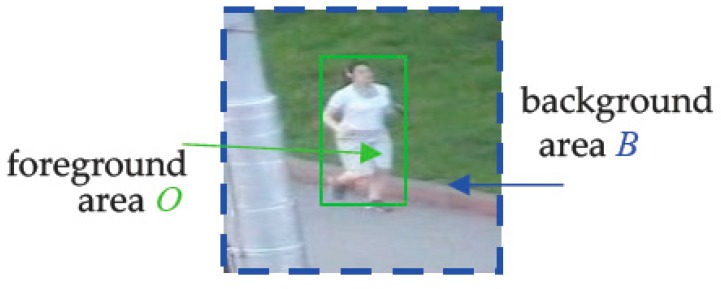
Training patch $T_{d,t}$. The region inside the green solid line is the foreground area O. The region which is outside of the green box (solid line) and inside of the blue box (dashed line) is the background area B.

As shown in Figure 2, during the model training phase in frame t, given as a rectangular patch $T_{d,t}$ which is sampled around the estimated location from frame t, Staple divided $T_{d,t}$ into the foreground area O (shares the size with the estimated target of previous frame) and background area B. Additionally, they were used to calculate the proportion of each index feature (32 × 32 × 32 bins space) in the foreground area O and the background area B, respectively. Suppose Ω is a region, Ω ∈ {O,B}, the proportion of each index feature j (32 × 32 × 32 bins space) in area Ω can be represented by $\rho^{j}\left(\Omega \right)=N^{j}\left(\Omega \right)/\left|\Omega \right|$, where $N^{j}\left(\Omega \right)=\left|\left\{u\in \Omega :\phi \left(u\right)=j\right\}\right|$ represents the number of index feature j in area Ω and |Ω| represents the total number of pixels in area Ω. Therefore, for an online model, $\rho^{j}\left(O\right)$ and $\rho^{j}\left(B\right)$ can be followed by the following formula:

(7) $$\begin{array}{l} \rho_{t}\left(O\right)=\left(1-\eta_{c}\right)\rho_{t-1}\left(O\right)+\eta_{c}\rho_{t}^{\prime }\left(O\right)\\ \rho_{t}\left(B\right)=\left(1-\eta_{c}\right)\rho_{t-1}\left(B\right)+\eta_{c}\rho_{t}^{\prime }\left(B\right) \end{array}$$

where $\rho_{t}\left(A\right)$ is the vector of $\rho_{t}^{j}\left(A\right),j=1,\ldots M.$ M is the dimension of the mapped space (32 × 32 × 32 bins), and $\eta_{c}$ is a learning rate parameter.

When calculating the proportion of each index feature j in the foreground area O and background area B, respectively, the weight coefficient $\beta_{t}^{j}$ for each index feature j is updated by the following equation:

(8) $$\beta_{t}^{j}=\frac{\rho_{t}^{j}\left(O\right)}{\left(\rho_{t}^{j}\left(O\right)+\rho_{t}^{j}\left(ℬ\right)+\lambda \right)},\;j=1,\ldots M.$$

**Figure 3:**
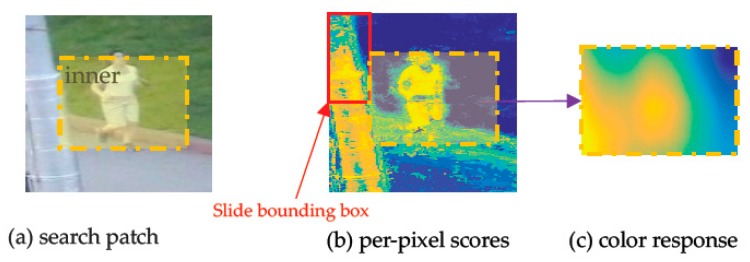
The color response calculation process of color model.

Given a RGB search patch $d_{t+1}$ (shown in Figure 3a) in frame *t* + 1, first, per-pixel scores $S_{t+1,\beta }$ (shown as heat map in Figure 3b) of search patch $d_{t+1}$ is obtained by looking up the table. Then, the score matrix $y_{t+1}^{c}$ named color response (shown as heat map in Figure 3c) of inner region r is obtained. The dotted yellow box region of search patch $d_{t+1}$ represents the inner region r, and the red box used to generate all of the boxes center at inner region r of $d_{t+1}$ in Figure 3b is the slide bounding box Π.

Given a search image $d_{t+1}$ = $\left[\begin{array}{cccc} u_{11} & u_{12} & \cdots & u_{1\hat{l}}\\ u_{21} & u_{22} & & \\ \vdots & & \ddots & \\ u_{\hat{c}1} & & & u_{\hat{c}\hat{l}} \end{array}\right]$, which (with a magnification relative to target bounding box $p_{t}$) is extracted around $p_{t}$ and has a size of $\hat{c}\times \hat{l}$, as well as the target size $s_{t}$, which is given for fixed-size target detection, we can obtain its inner region r and a bounding box set $ϒ=\left\{\Pi :\;l_{i,j}\left(\Pi \right)\;\in \;r,\;\vartheta \left(\Pi \right)=s_{t}\right\}$ corresponds to r in frame *t* + 1. From Equation (2), we know that our goal is to calculate the response color scores of all bounding boxes in ϒ. However, first we need to calculate a score matrix which represents the score of different pixels u in search image patch $d_{t+1}$, and the score matrix is also named per-pixel scores in Staple. The calculation process is as follows.

From the above training process, we know that the score (weights $\beta_{t}^{j}$) of each index feature j has been obtained in the previous frame t, therefore the score $\beta_{t}^{\phi \left(u\right)}$ of each pixel u (RGB space) in $d_{t+1}$ is obtained directly by looking it up in the table, therefore the score matrix $S_{t+1,\beta }$ = $\left[\begin{array}{cccc} \beta_{t}^{\phi \left(u_{11}\right)} & \beta_{t}^{\phi \left(u_{12}\right)} & \cdots & \beta_{t}^{\phi \left(u_{1l}\right)}\\ \beta_{t}^{\phi \left(u_{21}\right)} & \beta_{t}^{\phi \left(u_{22}\right)} & & \vdots \\ \vdots & & \ddots & \\ \beta_{t}^{\phi \left(u_{c1}\right)} & \cdots & & \beta_{t}^{\phi \left(u_{cl}\right)} \end{array}\right]$ named per-pixel scores is formed. The example of a per-pixel score is shown as the heat map in Figure 3b.

Then, we begin to calculate the color score of each bounding box Π in set ϒ. The color score of bounding box Π is a pixel-based average score, that is, the color score ($S_{c}(\Pi )$) of each bounding box Π is the average of the weight scores of all the pixels $\left(\left\{\beta_{t}^{\phi \left(u\right)},\;u\in \Pi \right\}\right)$ in the bounding box Π. The calculation formula is as follow:

(9) $$S_{c}(\Pi )=\frac{1}{\left|\Pi \right|}\sum_{u\in \Pi }\beta_{t}^{\phi \left(u\right)}$$

Then, by sliding the bounding box with fix-size $s_{t}$ on score matrix $S_{t+1,\beta }$, we can calculate all the color scores of bounding boxes in ϒ with Equation (9). Therefore, the other score matrix $y_{t+1}^{c}$ = $\left[\begin{array}{ccc} S_{c}\left(1,1\right) & \cdots & S_{c}\left(1,{\hat{w}}_{p}\right)\\ \vdots & \ddots & \\ S_{c}\left({\hat{h}}_{p},1\right) & & S_{c}\left({\hat{h}}_{p},{\hat{w}}_{p}\right) \end{array}\right]$ is obtained. Element $S_{c}\left(i,j\right)$ in $y_{t+1}^{c}$ represents the color score of bounding box centered at position (i,j) in inner region r, which is computed in Equation (9). $y_{t+1}^{c}$ shares the same size ${\hat{h}}_{p}\times {\hat{w}}_{p}$ with inner region r and is shown with the heat map in Figure 3c. Additionally, supposing the size of $s_{t}$ is ${\hat{h}}_{s}\times {\hat{w}}_{s}$, we have ${\hat{h}}_{p}=\hat{h}-{\hat{h}}_{s}+1$ and ${\hat{w}}_{p}=\hat{w}-{\hat{w}}_{s}+1$.

The fractional response of the sliding window can be accelerated by convolving the image in Staple. For more details, one can refer to the code of the Staple algorithm.

### 2.3. Learning of Object Model

Similar to the filter model and color model, the object model is also based on the tracking-by-detection principle to locate the target. In Section 2.2, we find that, in the color model, the score of each bounding box is calculated based on the average of all pixel weight scores in the bounding box. In this section, we describe another approach, which is based on contour information to measure the probability score of the bounding box as a target.

In Edge Boxes [18], the likelihood of the bounding box containing an object is based on the number of contours that are wholly contained in a bounding box. Using efficient data structures, millions of bounding boxes can be evaluated in a fraction of a second. Furthermore, this model does not require additional training process; given the location and size of bounding box and the image, the score of the target box can be calculated efficiently. Edge Boxes [18] is introduced below.

**Figure 4:**
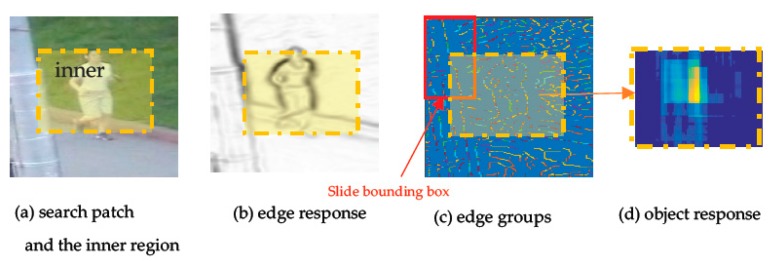
The calculation process of object model. For a given search patch $d_{t+1}$ (**a**), first, the edge response (**b**) and the edge groups (shown with the heat map in (**c**)) of search area $d_{t+1}$ is computed with the method in Edge Boxes [18], respectively. Finally, the object response $y_{t+1}^{o}$ (shown with the heat map in (**d**)) of inner region r is obtained, which represents the scores of all the boxes in set ϒ. The red box in (**c**) is the slide bounding box used to generate the center of all the bounding boxes at inner region r of $d_{t+1}$.

Given a search area $d_{t+1}$, which (with a magnification relative to target bounding box $p_{t}$) is extracted around $p_{t}$ and has size of $\hat{c}\times \hat{l}$, as well as the target size $s_{t}$, which is given for a fixed-size target detection, similar to the color model, we can obtain its inner region r and a bounding box set $ϒ=\left\{\Pi :\;l_{i,j}\left(\Pi \right)\in r,\;\vartheta \left(\Pi \right)=s_{t}\right\}$ that corresponds to r in frame t+1. Again, similar to the color model, our goal is to calculate the response scores of all bounding boxes in ϒ. However, before calculating the scores of the bounding boxes in ϒ, we should obtain the edge response and edge groups of search area $d_{t+1}$ in turn, with the method in Edge Boxes. Examples of edge response and edge groups are shown in Figure 4b,c, respectively. Specific calculations can be found in Edge Boxes.

After all the edge groups have been calculated, from Edge Boxes, we know that the object score $S_{o}\left(\Pi \right)$ of each bounding box Π can be expressed as:

(10) $$S_{o}\left(\Pi \right)=\frac{\sum_{i}\varsigma_{i}m_{i}}{2{\left(b_{w}+b_{h}\right)}^{\varpi }}-\frac{\sum_{r\in b^{in}}m_{r}}{2{\left(b_{w}+b_{h}\right)}^{\varpi }}$$

where $b_{w}$ and $b_{h}$ are the width and height of the bounding box Π, respectively; $b^{in}$ represents a central region of the Π; r is an edge (corresponding to a pixel) which has an edge magnitude $m_{r}$, and $m_{i}$ is the sum of the edge magnitude $m_{r}$ for all edges r in ${\overset{⌢}{g}}_{i}$; $\varsigma_{i}\in [0,1]$ is a continuous value to indicate the probability that ${\overset{⌢}{g}}_{i}$ belongs to a fixed bounding box Π; and ϖ is the penalty coefficient of the size of Π. For more details, one can refer to the code of the Edge Boxes algorithm.

Therefore, we can calculate the object scores of each bounding box in ϒ by sliding the bounding box. Then, the other score matrix $y_{t+1}^{o}$ = $\left[\begin{array}{ccc} S_{o}\left(1,1\right) & \cdots & S_{o}\left(1,{\hat{w}}_{p}\right)\\ \vdots & \ddots & \\ S_{o}\left({\hat{h}}_{p},1\right) & & S_{o}\left({\hat{h}}_{p},{\hat{w}}_{p}\right) \end{array}\right]$, named the object response of inner region r, is obtained, which shares the same size ${\hat{h}}_{p}\times {\hat{w}}_{p}$ with inner region r and is shown with the heat map in Figure 4d, where $S_{o}\left(i,j\right)$ computed with Equation (10) is the object score of a bounding box in ϒ, which has the position (i,j) in region r and the size of $s_{t}$.

### 2.4. Final Response Scores Calculation of MMT

**Figure 5:**
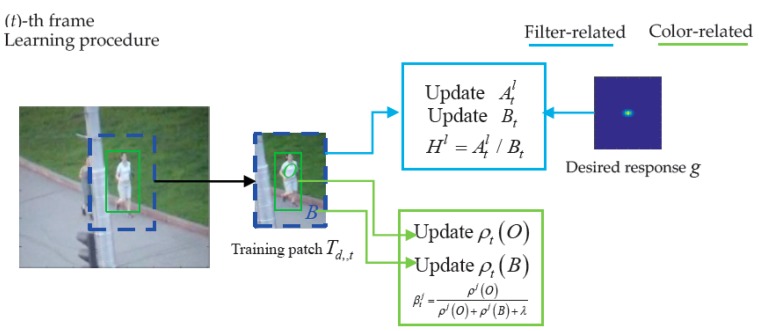
Transition tracking framework of multi-complementary model tracker (learning procedure). In the (t)-th frame, a training patch is extracted at the location of for updating the denominator, and the numerator of the translation filter with Equation (5). The target’s foreground area and background area are obtained from, and are used to calculate the proportion vectors and with Equation (7), respectively. Then, the score (weights) of each index feature is updated in Equation (8).

**Figure 6:**
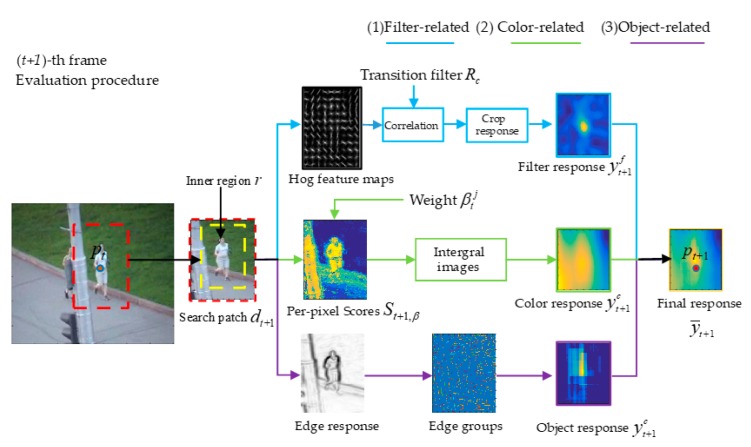
Transition tracking framework of multi-complementary model tracker (evaluation procedure).

Given a search area $d_{t+1}$, which has size of $\hat{c}\times \hat{l}$, and the fixed target size $s_{t}$ of the previous frame, similar to the color and object models, we can obtain its inner region r and a bounding box set $ϒ=\left\{\Pi :\;l_{i,j}\left(\Pi \right)\in r,\;\vartheta \left(\Pi \right)=s_{t}\right\}$ that corresponds to r in frame *t* + 1. From the above, we know that we can obtain three scores including the color score $S_{c}(\Pi )$, filter score $S_{f}(\Pi )$, and object score $S_{o}(\Pi )$ computed by the color model, filter model, and object model, respectively, and the three response scores all represent the possibility of all the bounding boxes (with the fixed size of $s_{t}$ and contains our search space) centered at different positions in inner region r by different model parameters. Since the three response scores were all between 0 and 1 (1 to the object and 0 to other), the magnitude of the response scores was compatible. Therefore, we followed the Staple algorithm and fused the three response scores by linear weighting. For each Π∈ϒ, we obtained three scores $S_{c}(\Pi )$, $S_{o}(\Pi )$, and $S_{f}(\Pi )$ from three models, respectively. Finally, according to Equation (2), the final fusing score of bounding box Π was calculated by weighting $S_{c}(\Pi )$, $S_{o}(\Pi )$ and $S_{f}(\Pi )$ as:

(11) $$\bar{S}(\Pi )=\left(\tau_{c}\right)S_{c}(\Pi )\;+\;\left(\tau_{o}\right)S_{o}(\Pi )\;+\;\left(1-\tau_{c}-\tau_{o}\right)S_{f}(\Pi )$$

where $\tau_{c}$ and $\tau_{o}$ are the merge coefficients of the score of the color model and object detection, respectively, and the sizes set in the experiment were 0.2 and 0.25, respectively. Specific parameters of the experiment are presented below. When the fusing scores of all the bounding boxes centered in inner region r are computed, a fusing scores matrix ${\bar{y}}_{t+1}$ = $\left[\begin{array}{ccc} \bar{S}\left(1,1\right) & \cdots & \bar{S}\left(1,{\hat{w}}_{p}\right)\\ \vdots & \ddots & \\ \bar{S}\left({\hat{h}}_{p},1\right) & & \bar{S}\left({\hat{h}}_{p},{\hat{w}}_{p}\right) \end{array}\right]$, named the final response, is obtained, where $\bar{S}\left(i,j\right)$, computed with Equation (11), is the fusing score of a bounding box in ϒ, which has the position (i,j) in region r and the size of $s_{t}$. After selecting the target bounding box $p_{t+1}$ with the largest corresponding score of the final response ${\bar{y}}_{t+1}$, the target scale is calculated by the scale filter. The transition tracking part of the MMT algorithm, which incorporates the object model to Staple, is shown in Figure 5 (learning procedure) and Figure 6 (evaluation procedure).

From Equation (2), we know that $p_{t}$ denotes the estimated position of the target in (*t*)-th frame and $p_{t+1}$ denotes the predicted position of the target in the (*t +* 1)-th frame. Given the search patch $d_{t+1}$, which (with a magnification relative to target bounding box $p_{t}$) is extracted around the $p_{t}$, and the size $s_{t}$ of the target in the previous frame, we can obtain inner region r, $r\subset d_{t+1}$, and a box set ϒ, which is our search sample space. The purpose of the transition tracking of the multi-complementary model is to choose the new target bounding box $p_{t+1}$ with max fusing score from the box set ϒ. Therefore, after the final response ${\bar{y}}_{t+1}$, which gives the fusing scores of all the boxes in ϒ calculated with Equation (11), $p_{t+1}$ is estimated at the peak of ${\bar{y}}_{t+1}$. When the new position is obtained, the scale of target is computed by the 1D correlation filter. Specific details about scale estimation can be found in the code of Staple. $y_{t+1}^{f}$, $y_{t+1}^{c}$, and $y_{t+1}^{o}$ are computed by the filter model, color model, and object model, respectively. These are used to obtain final response ${\bar{y}}_{t+1}$ with Equation (11) and their calculation process will be explained below.

(1) Filter-related. In the (*t* + 1)-th frame, the search patch $d_{t+1}$ (with a magnification relative to target $p_{t}$) is extracted around the $p_{t}$, and the search feature maps represented using HOG features are extracted from $d_{t+1}$ and then convolved with translation filter $R_{C}$ through Equation (6) to calculate the filter response of $d_{t+1}$. Due to inner region $r\subset d_{t+1}$, the filter response $y_{t+1}^{f}$ of inner region r can be obtained by cropping the filter response (score matrix) of $d_{t+1}$. $y_{t+1}^{f}$ is a matrix which takes the scores of all the bounding boxes (with the same size of $s_{t}$, $\vartheta \left(\Pi \right)=s_{t}$) centered at different positions in inner region r as elements.
(2) Color-related. In the (*t* + 1)-th frame, the per-pixel scores $S_{t+1,\beta }$, which represent the scores of pixels at different positions of $d_{t+1}$, are obtained by looking up the table of weight $\beta_{t}^{j}$, then the color response $y_{t+1}^{c}$, which represents the color scores of all the bounding boxes in set ϒ, is obtained with Equation (9).
(3) Object-related. In the (*t* + 1)-th frame, we calculate the edge response and the boundary group for the search area $d_{t+1}$ in turn with the method in Edge Boxes [18]. Then, the object response $y_{t+1}^{o}$, which represents the object scores of all the bounding boxes in set ϒ, is calculated with Equation (10).

### 2.5. Scale Calculation Optimization

When target bounding box $p_{t+1}$, which denotes the predicted position of the target in frame t+1, is obtained by translation tracking, the object scale can be calculated using the one-dimensional scale filter proposed in [15]. The size range selection principle is as follows:

(12) $$\kappa^{n}w_{t-1}*\kappa^{n}h_{t-1},\;n\in \left\{-\left(\nu -1\right)/2,\ldots ,\left(\nu -1\right)/2\right\}$$

where $w_{t-1}$, $h_{t-1}$ are the width and height of the object on the previous frame, respectively; κ is the scale factor; and ν is the scale number. We followed Staple and set κ and ν to 1.02 and 33, respectively.

Due to the classical scale calculation method in [15] (which is also used in Staple), we needed to calculate the HOG feature maps of 33 scale image blocks during training and testing, which is very complicated. In frame t, the feature map of the scale testing and the scale training were all based on the same coordinates, which were obtained from the transition tracking of frame t. The scale testing needs to extract the 33-scale image patch features relative to the target scale of the (*t* − 1)-th frame and scale training needs to extract the 33-scale image patch features relative to the target scale of the (*t*)-th frame, respectively, as the two-frame scale change is usually small or even the same. In the case where the scale of the two frames before and after the change is n, the image features of the 33−n sample blocks are repeatedly calculated, resulting in significant complexity. In this paper, we therefore reused the features of the scale image patch that were obtained in the process of doing the scale calculations during scale updating. This optimization method greatly improved the execution speed of the tracker.

## 3. Multi-Complementary Model for Long-Term Tracking

With the MMT tracker and the new proposed detection method, we constructed the MMLT (Multi-Complementary Model for Long-term Tracking) tracker. In the following, we introduce the online detection module in detail. In Section 3.1, we describe the proposed online detector method used to get the candidate bounding boxes. In Section 3.2, we present how we evaluated the confidence score of the candidate bounding box. In Section 3.3, we present how we obtained the redetected target and how we decided whether to use it to reinitialize the tracker. In Section 3.4, we introduce how the MMT tracker and online detection module worked together in MMLT comprehensively and introduce the detection module’s high confidence update mechanism in this paper.

### 3.1. The Online Detector

It is common sense that the detection module is necessary for a long-term tracking method to redetect the target in case of failed tracking when long-term occlusion or out-of-view arise. In addition, the detection method of learning a classifier online and using a classifier to search by sliding the window has high time complexity. Different from previous works [26,30] where the online classifier needs to be trained, in this paper, we combined an object detection method based on object contour [18] and the color detection method [25] to generate a fractional prediction response of the search area to redetect the target ${\tilde{p}}_{t+1}$. Due to the diversity of the sample, information and discriminant can be utilized to a greater extent. By integrating the dual prediction scores, we could form the fusing prediction response score for an object with greater confidence, resulting in better detection. This greatly improves the generalizability of the tracker. 

Give a detection area $X_{t+1}$ = $\left[\begin{array}{cccc} {\tilde{u}}_{11} & {\tilde{u}}_{12} & \cdots & {\tilde{u}}_{1\hat{c}}\\ {\tilde{u}}_{21} & {\tilde{u}}_{22} & & \vdots \\ \vdots & & \ddots & \\ {\tilde{u}}_{\hat{a}1} & \cdots & & {\tilde{u}}_{\hat{a}\hat{c}} \end{array}\right]$, which (with a magnification relative to target $p_{t}$) is extracted around $p_{t}$, it has a size of $\tilde{a}\times \tilde{c}$ and the fix target size $s_{t}$ obtained from the previous frame. Similar to the tracking module, we can also obtain its inner region $\tilde{r}$ and a bounding box set $\tilde{ϒ}=\left\{\Pi :\;l(\tilde{\Pi })\in \tilde{r},\;\vartheta \left(\tilde{\Pi }\right)=s_{t}\right\}$ corresponding to $\tilde{r}$. In addition, our goal is still to calculate the response scores of all bounding boxes in $\tilde{ϒ}$ by the color and object parameters.

For the object detection model, according to the method in Section 2.3, the edge response and edge groups of $q_{o,t+1}$ are computed in turn. Then, we obtain object response $d_{o,t+1}=$ $\left[\begin{array}{ccc} S_{o}\left(1,1\right) & \cdots & S_{o}\left(1,{\tilde{w}}_{p}\right)\\ \vdots & \ddots & \vdots \\ S_{o}\left({\tilde{h}}_{p},1\right) & \cdots & S_{o}\left({\tilde{h}}_{p},{\tilde{w}}_{p}\right) \end{array}\right]$ with Equation (10), which presents the object scores of all the bounding boxes centered in inner region $\tilde{r}$. At the same time, the per-pixel scores matrix $S_{t+1,\tilde{\beta }}$ = $\left[\begin{array}{cccc} {\tilde{\beta }}_{t}^{\phi \left({\tilde{u}}_{11}\right)} & {\tilde{\beta }}_{t}^{\phi \left({\tilde{u}}_{12}\right)} & \cdots & {\tilde{\beta }}_{t}^{\phi \left({\tilde{u}}_{1\tilde{c}}\right)}\\ {\tilde{\beta }}_{t}^{\phi \left({\tilde{u}}_{21}\right)} & {\tilde{\beta }}_{t}^{\phi \left({\tilde{u}}_{22}\right)} & & \vdots \\ \vdots & & \ddots & \\ {\tilde{\beta }}_{t}^{\phi \left({\tilde{u}}_{\tilde{a}1}\right)} & \cdots & & {\tilde{\beta }}_{t}^{\phi \left({\tilde{u}}_{\tilde{a}\tilde{c}}\right)} \end{array}\right]$ is obtained using ${\tilde{\beta }}_{t}^{j}$, and the color response $d_{c,t+1}$ = $\left[\begin{array}{ccc} S_{c}\left(1,1\right) & \cdots & S_{c}\left(1,{\tilde{w}}_{p}\right)\\ \vdots & \ddots & \vdots \\ S_{c}\left({\tilde{h}}_{p},1\right) & \cdots & S_{c}\left({\tilde{h}}_{p},{\tilde{w}}_{p}\right) \end{array}\right]$ that represents the color scores of bounding boxes (with the same size of $s_{t}$) centered at different positions in inner region $\tilde{r}$ is also computed efficiently through the integral images (Equation (9)).

After we obtain the predicted response scores of different models, the approach of fusing the predictions of the multi-complementary detection model is as follows. 

Let us assume that the prediction results of different models are independent. $\tilde{\Pi }\in \tilde{ϒ}$ is chosen from a box set $\tilde{ϒ}$, and υ∈{−, +} denotes a foreground-background label. ${\tilde{M}}_{\theta }$ is the parameter corresponding with different models, which depends on the previous object state and previous frame. The likelihood of the candidate bounding box $\tilde{\Pi }$ belongs to the object under the model parameter ${\tilde{M}}_{\theta }$, which can be expressed as $p\left(\tilde{\Pi }=+;{\tilde{M}}_{\theta }\right),\theta =1,\ldots n$, where n is the total number of models. Suppose the models are independent of each other, then we have the following decomposition:

(13) $$p\left(\tilde{\Pi }=+;{\tilde{M}}_{1}\ldots {\tilde{M}}_{\theta }\ldots {\tilde{M}}_{n}\right)=\prod_{\theta }p\left(\tilde{\Pi }=+;{\tilde{M}}_{\theta }\right),\theta =1,\ldots n$$

Now, we have two independent model parameters: the color model parameter ${\tilde{M}}_{0}$ and the object model parameter ${\tilde{M}}_{1}$. According to the color model parameter ${\tilde{M}}_{0}$, the probability that candidate bounding box $\tilde{\Pi }$ is the object can be calculated by

(14) $$p\left(\tilde{\Pi }=+;{\tilde{M}}_{0}\right)=S_{o}\left(\tilde{\Pi }\right)$$

Likewise, according to the object detection model parameter ${\tilde{M}}_{1}$, the probability that the candidate bounding box $\tilde{\Pi }$ is the object can be calculated by

(15) $$p\left(\tilde{\Pi }=+;{\tilde{M}}_{1}\right)=S_{c}\left(\tilde{\Pi }\right)$$

Therefore, according to Equation (13), we have the following formula to fuse the predictions of the different independent models for calculating the score of each bounding box $\tilde{\Pi }$:

(16) $$p\left(\tilde{\Pi }=+;{\tilde{M}}_{0},{\tilde{M}}_{1}\right)=S_{o}\left(\tilde{\Pi }\right)\times S_{c}\left(\tilde{\Pi }\right)$$

When we get the scores of all the bounding boxes in $\tilde{ϒ}$, from Equation (16), we know the final response matrix ${\bar{d}}_{t+1}$ can be obtained by:

(17) $${\bar{d}}_{t+1}=d_{o,t+1}⊙d_{c,t+1}$$

where ⊙ represents dot multiplication operations. ${\bar{d}}_{t+1}$ represents the final response scores of all the bounding boxes $\tilde{\Pi }$ (with the same size of $s_{t}$, $\vartheta \left(\tilde{\Pi }\right)=s_{t}$) centered at different positions in the inner region $\tilde{r}$.

In general, the probability of $\tilde{\Pi }$ to the object is large when both the color score and the object detection score of $\tilde{\Pi }$ are high. If the $\tilde{\Pi }$ gets a low score from any one model, it is considered as not likely to be the target. Therefore, in this paper, the color score and the object detection score are merged by multiplication to obtain more reliable detection results. 

Having calculated the final response ${\bar{d}}_{t+1}$ of inner region $\tilde{r}$ (in detection area $X_{t+1}$), the corresponding peaks of ${\bar{d}}_{t+1}$ could be used to obtain the possible object location. Then, the purpose of the detector is to detect the top-w (w = 10) confident detection bounding boxes from $\tilde{ϒ}$ (corresponding to inner region $\tilde{r}$). First, we select the bounding box $l_{\max}$ centered at the peak (with the largest response score $M_{s}$ in the final response ${\bar{d}}_{t+1}$) to the candidate bounding boxes set T. When the ratio between the response score of the bounding box centered at other peaks to $M_{s}$ is greater than a threshold ξ, the corresponding bounding box is also added to T. Similar to CCT, the bounding box $l_{0}$ centered at the position in the previous frame is also added to T as a candidate bounding box for evaluation. At the same time, we limited the total number of candidate bounding boxes so that it does not exceed w = 10. Finally, we obtain the candidate bounding box set $T=\left\{l_{0},l_{1},l_{2},\cdots ,l_{k}\right\}$.

### 3.2. Candidate Bounding Box Evaluation

After the detection module detects the candidate bounding box set T, a robust mechanism is needed to measure the confidence score of each candidate bounding box $l_{i}\in T$. However, to effectively measure the confidence score of each bounding box, we follow the Collaborative Correlation Tracker (CCT) [31] and consider not only the target area corresponding to the bounding box, but also the information of its background area. That is, for each candidate bounding box $l_{i}$, we extract the image region samples EB-patch ${\tilde{l}}_{i}$, which centers at the location of $l_{i}$ and has the same magnification relative to the candidate bounding box as transition filter $R_{c}$. The EB-patch ${\tilde{l}}_{i}$ is measured by a well-trained filter ${\tilde{R}}_{c}$ (similar to $R_{c}$) to obtain the confidence score ${\tilde{s}}_{i}$ of the corresponding candidate bounding box $l_{i}$.

First, we obtain the candidate EB-patch set $S=\left\{{\tilde{l}}_{0},\;{\tilde{l}}_{1},\;{\tilde{l}}_{2}\cdots {\tilde{l}}_{k}\right\}$ corresponding to $T=\left\{l_{0},\;l_{1},\;l_{2}\cdots l_{k}\right\}$. For each EB-patch ${\tilde{l}}_{i}\in S,i\in \left(0,1,\cdots ,k\right)$, its HOG features map $Jl_{i}$ is calculated, which convolved with confidence filter ${\tilde{R}}_{c}$ in Equation (6), to obtain the confidence filter response ${\hat{y}}_{i}$. In addition, we used the maximum score ${\tilde{s}}_{i}=\max\left({\hat{y}}_{i}\right)$ of ${\hat{y}}_{i}$ as the confidence score of the candidate bounding box $l_{i}$. Finally, the candidate confidence scores $\tilde{S}=\left\{{\tilde{s}}_{0},{\tilde{s}}_{1},{\tilde{s}}_{2}\cdots {\tilde{s}}_{k}\right\}$ are also obtained. The calculation of ${\tilde{R}}_{c}$ is given in Section 3.3.

### 3.3. Redetected Result Decision

When the candidate confidence scores $\tilde{S}$ is obtained, the final redetected target can be calculated as:

(18) $$\overset{⌢}{i}=\underset{i}{\arg\max}\left(\delta *{\tilde{s}}_{0},{\tilde{s}}_{1},{\tilde{s}}_{2}\cdots {\tilde{s}}_{k}\right)$$

Then, the bounding box $l_{\overset{⌢}{i}}$ is the final redetected target ${\tilde{p}}_{t+1}$. When $\tilde{i}\neq 0$ and ${\tilde{s}}_{\tilde{i}}$ is higher than a certain threshold χ, ${\tilde{p}}_{t+1}$ is accepted, and is then used to initialize the tracker. When $\tilde{i}=0$ or ${\tilde{s}}_{\tilde{i}}$ is lower than χ, we consider ${\tilde{p}}_{t+1}$ is not correct, and it is not accepted. 

### 3.4. Multi-Complementary Model for Long-Term Tracking

**Figure 7:**
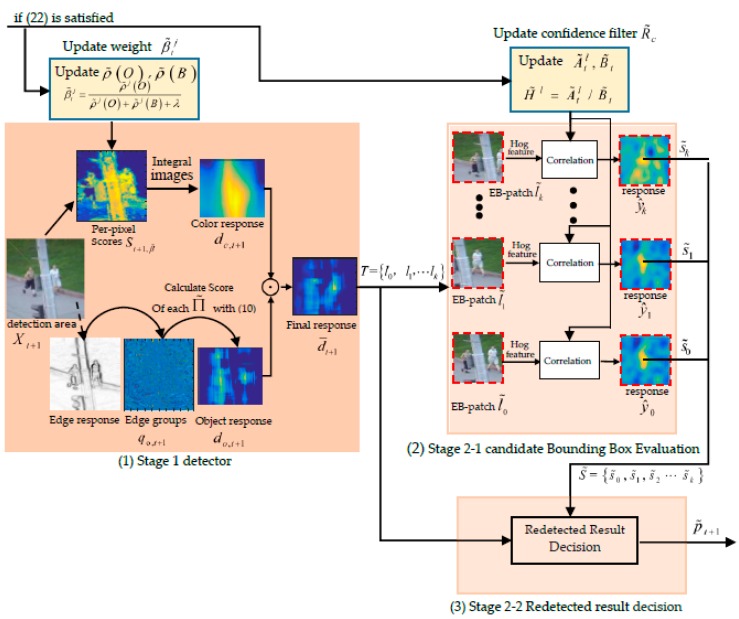
Online detection module based on complementary model response. ⊙ represents dot multiplication operations of matrix.

Clearly, a robust long-term tracking algorithm requires a re-detection module in case of tracking failure. Similar to LCT, we use the threshold $\tau_{m}$ as the activation confidence to activate the detector. First, MMT performs the target tracking process in each frame. When the activation confidence $\max(y_{t}^{h})<\tau_{m}$, the detector is activated to redetect the target, where $y_{t}^{h}$ is the filter response of the transition tracking, which is computed in Equation (6). For the detection process, first, the detector has to compute the possible candidate bounding box set $T=\left\{l_{0},\;l_{1},\;l_{2}\cdots l_{k}\right\}$, and then the candidate EB-patch set $S=\left\{{\tilde{l}}_{0},\;{\tilde{l}}_{1},\;{\tilde{l}}_{2}\cdots {\tilde{l}}_{k}\right\}$ corresponds to set T. Then, the confidence score set $\tilde{S}=\left\{{\tilde{s}}_{0},{\tilde{s}}_{1},{\tilde{s}}_{2}\cdots {\tilde{s}}_{k}\right\}$ is obtained by calculating the confidence score of each EB-patch in *S* with the confidence filter ${\tilde{R}}_{C}$ and Equation (6). Then, the redetected result decision module computes the redetected target ${\tilde{p}}_{t+1}$ and decides whether to use it to reinitialize the tracker. The overall procedure of the online detection module is shown in Figure 7.

Similar to LCT, our confidence filter model is only updated when the tracking results are reliable. LCT uses the response value of the confidence filter to measure the reliability of the tracking results. We follow LMCF (Large Margin Object Tracking with Circulant Feature Maps) [32] and considered whether the object tracking results are reliable depending on both the maximum response value and the response map’s APCE (Average Peak-to Correlation Energy) [32]. LCT trained an independent confidence filter to evaluate the confidence score of each candidate box in the detector. However, this adds extra time complexity to the algorithm. CCT uses the filter used in the tracking process to evaluate the confidence score of each candidate bounding box; thus, as the tracking model drifted, the detection model also drifted. Therefore, the reliability of the re-detection result obtained by the detection module could not be guaranteed. In this paper, we use a copy version ${\tilde{R}}_{c}$ of translation filter $R_{c}$ in the tracking process to measure each EB-patch to obtain the confidence score of each candidate bounding box; however, to prevent drift occurring in the detection model, as in the tracking model, different from $R_{c}$, which is updated each frame, the filter ${\tilde{R}}_{c}$ is updated only when the tracking result is reliable. Therefore, we update the numerator ${\tilde{A}}_{t}^{l}$ and denominator ${\tilde{B}}_{t}$ of the confidence filter ${\tilde{H}}_{t}^{l}$(${\tilde{R}}_{c}$) when Equation (22) is satisfied. The formula is as follows:

(19) $$\{\begin{array}{c} {\tilde{A}}_{t}^{l}=\left(1-\eta_{f}\right){\tilde{A}}_{t-1}^{l}+\eta_{f}\bar{G}F_{t}^{l}\;\mathrm{if}\;\mathrm{Equation}\;\left(22\right)\;\mathrm{is}\;\mathrm{satisfied}\\ {\tilde{A}}_{t}^{l}={\tilde{A}}_{t-1}^{l}\;\mathrm{else} \end{array}$$

(20) $$\{\begin{array}{c} {\tilde{B}}_{t}=\left(1-\eta_{f}\right){\tilde{B}}_{t-1}+\eta_{f}\overset{d}{\underset{k=1}{\sum }}\bar{F_{t}^{k}}F_{t}^{k}\;\mathrm{if}\;\mathrm{Equation}\;\left(22\right)\;\mathrm{is}\;\mathrm{satisfied}\\ {\tilde{B}}_{t}={\tilde{B}}_{t-1}\;\mathrm{else} \end{array}$$

(21) $${\tilde{H}}_{t}^{l}=\frac{{\tilde{A}}_{t}^{l}}{{\tilde{B}}_{t}}l=1,2,\cdots N.$$

where $\bar{G}F_{t}^{l}$ and $\overset{d}{\underset{k=1}{\sum }}\bar{F_{t}^{k}}F_{t}^{k}$ are polynomials that have been calculated in the updating of numerator $A_{t}^{l}$ and denominator $B_{t}$ of transition filter $R_{c}$ in the transition tracking process with Equation (5).

Following the updating mechanism of filter ${\tilde{R}}_{c}$, ${\tilde{\rho }}_{t}\left(O\right)\;\mathrm{and}\;{\tilde{\rho }}_{t}\left(B\right)$ are also the copy versions of $\rho_{t}\left(O\right)\;\mathrm{and}\;\rho_{t}\left(B\right)$ for detecting in the detector, which are updated with Equation (7) only when Equation (22) is satisfied. Similar to $\beta_{t}^{j}$, the updating of ${\tilde{\beta }}_{t}^{j}$ is in Equation (8). The high confidence update mechanism that represents a reliable tracking in detection model is as follows.

In the (*t*)-th frame, we follow LMCF [32] and use the maximum filter response value $y_{t}^{f}\left(pmax\right)=\max\left(y_{t}^{f}\right)$ and the APCE (Average Peak-to Correlation Energy) [32] of the filter response map $y_{t}^{f}$ as a reference for updating the model, where $y_{t}^{f}$ is the filter response of translational tracking phase, which is obtained by Equation (6) in the (*t*)-th frame. The calculation method of APCE can refer to LMCF. Then, we consider the object tracking results reliable when both criterion $y_{t}^{f}\left(pmax\right)$ and the response map’s APCE ${ℸ}_{l}$ are greater than their respective Δ(Δ=5) frames historical average values ${\tilde{y}}_{t}^{f}\left(pmax\right)$ and ${ℸ}_{t}^{*}$. Thus, whether the target tracking result in the current frame is reliable can be judged by whether the following conditions are satisfied:

(22) $$\begin{array}{l} y_{t}^{f}\left(pmax\right)\;\geq \;\omega_{1}*{\tilde{y}}_{t}^{f}\left(pmax\right)\\ {ℸ}_{t}\;\geq \;\omega_{2}*{ℸ}_{t}^{*} \end{array}$$

## 4. Performance Evaluation

We implemented our experiment on the OTB-13 [33] and OTB-15 [34] benchmark datasets. All of the video sets with challenging factors were selected to undertake three experiments to evaluate performance: one pass evaluation (OPE), temporal robustness evaluation (TRE), and spatial robustness evaluation (SRE). OPE uses a traditional method of evaluation. As pointed out by [33], the traditional one-pass evaluation cannot fully reflect the robustness of a tracker, and sometimes even a small disturbance can lead to very different tracking results. The tracker begins tracking at the true position of the initial frame and calculates the Precision and Success Rate (SR). The TRE and SRE are different: the TRE randomizes the start frame to run the tracker on the rest of the sequence; and the SRE verifies the performance of a tracker by tracking an object after shifting and scaling the initial object box. These three kinds of evaluation monitor performance by indicating the accuracy and success rate of the generated graph, which indicates the percentage of the number of frames that the tracker has been able to track under different thresholds. 

In this section, we first evaluate MMLT with the improvements from the online detector and multi-complementary model on OTB-13. Then, we compare MMLT with nine of the most related and state-of the-art trackers on the OTB-13 and OTB-15 benchmark datasets. Finally, we analyze the effect of different merge parameters on the tracking performance. All of the tracking results used the reported results to ensure a fair comparison.

### 4.1. Experimental Configuration and Parameter Settings

To evaluate the performance and efficiency of the proposed algorithm, our tracker was implemented in Matlab software with a Core i7 4.0GHz CPU and 8GB RAM. MMLT runs faster than 50 frames per second (FPS). The color model used a 32-bit RGB channel color model. HOG features were selected as the features of interest, the cell size was set to four, and the number of statistical gradient directions was nine. As in [25], the translation filter image block parameter had a fixed area to achieve standardization. The parameter was set to 150 × 150.

We, following Staple, only searched in the area around the previous position for both translation and scale in the tracking module, and adopted the translation filter $R_{C}$ scale filter for tracking using a Hann window during the search as well as for training. Additionally, also following the Staple, we normalized the translation search patch by a parameter’s fixed area, and weighted the extracted feature channels patch of the target and context by a cosine window. Thirty-three scales with a scale factor of 1.02 were used in the scale model in this paper. In this paper, all search patches extracted around the previous position included both the target and surrounding context. In addition, we also adopted the confidence filter ${\tilde{R}}_{C}$ in the candidate detection EB-patch evaluation using a Hann window during confidence evaluation as well as for training and normalized the EB-patch by the parameters fixed area. The specific parameters can be seen in Table 1. More detailed parameters setting about the Staple algorithm can be seen in the code of Staple.

### 4.2. Analysis of MMLT Improvement

To validate the tracking aspect of the object detection and the effectiveness of the detection module, we also compared several baselines of MMLT on the OTB-13 with all 51 videos. The MMLT-NN in Table 2 is an accelerated version of the Staple algorithm that incorporated our scale optimization. We named the tracker that had an object model in transition tracking but had no online detector module as MMT. The tracker that had no object model in transition tracking but had an online detector module was named MMLT-N1. The specific tracking performance in the OTB-13 benchmark datasets are shown in Table 2, where the average FPS is the average FPS for the tracker. To more fully measure the execution efficiency of the tracking algorithm, the tracking speed of the tracker listed in Table 2 was the average of the tracking speed of 51 videos in the OTB-13 benchmark datasets. It is worth noting that the performance in Table 2 of the Staple algorithm was based on the source code provided by the author and the parameters given in Staple [25]. 

As seen in Table 2, MMLT’s tracking accuracy and tracking success rate were superior to all other tracker versions in all three metrics of OPE, TRE, and SRE. At the same time, it was seen that the average tracking accuracy and the average success rate of MMLT had more than 10% hits against with Staple, and real-time tracking of 50 FPS was achieved at the same time. For Staple, the tracking performance was much weaker than other versions of trackers in this paper since only the filter model and the color model were integrated into the translation tracking stage and there was no detection model. As shown in Table 2, the MMLT-NN algorithm with the tracking speed of 135 FPS was greatly improved when compared to Staple due to the optimization of scale calculation. At the same time, MMT had an average of 6% and an average of over 7% improvement in tracking precision and tracking success rate, respectively, when compared with Staple due to the inclusion of the object detection model during the translational tracking phase. The MMLT-N1 tracker also had good performance due to the detection mechanism added in the case of tracking failure. Compared with the Staple, the MMLT-N1 tracker also improved the tracking precision and tracking success rate by 6% and 7%, respectively. At the same time, in Table 2, we can see that the tracking speed of the MMLT-N1 was lower than that of the MMLT, which adopted the target detection model and the object model in transition tracking. Without the object model in transition tracking, the tracking robustness of MMLT-N1 was relatively low, and the target tracking was prone to drift, which led to the MMLT-N1 tracker needing more re-detection processes and a decrease in tracking speed. At the same time, it could indirectly reflect the importance of integrating the object detection model in the translation tracking phase. In summary, from the experimental results, the multi-complementary model tracking algorithm proposed in the translation phase or the proposed target re-detection algorithm both provided a significant improvement in the performance of the algorithm.

### 4.3. MMLT Experiment

**Figure 8:**
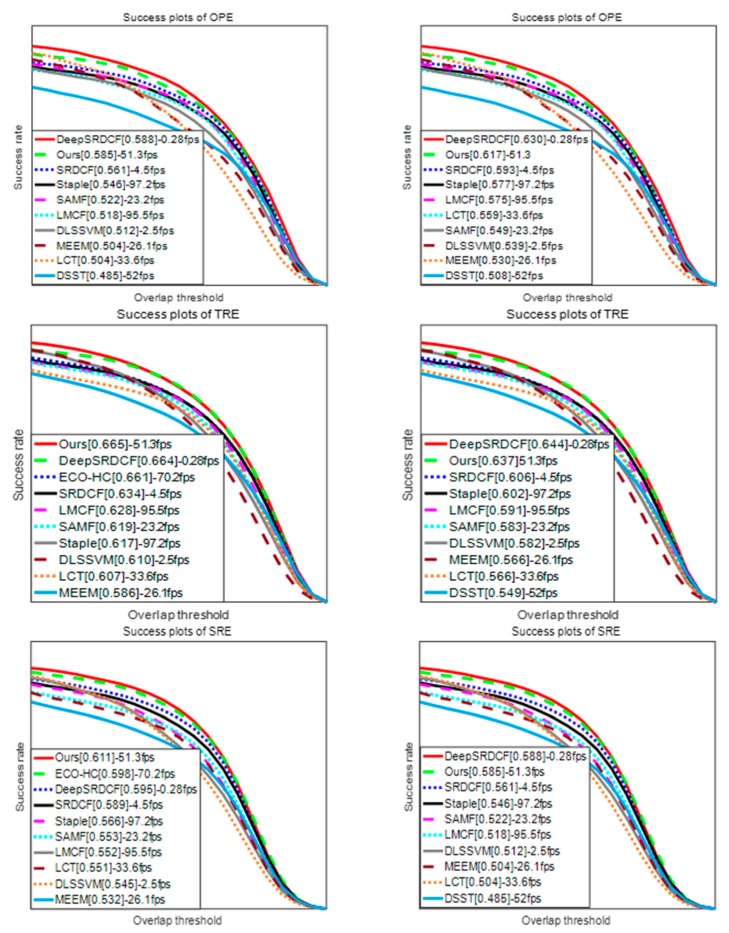
The success plots of OPE, TRE, and SRE on: OTB-13 (**left**); and OTB-15 (**right**). The left scores in the legend represent the scores of the corresponding indicators relating to the different tracking algorithms. The right scores in the legend represent the average FPS of the different trackers.

We compared our algorithm with some state-of-the-art methods including, SRDCF (Spatially Regularized Discriminative Correlation Filter Tracker [7]), DeepSRDCF (Spatially Regularized Discriminative Correlation Filter Tracker Based Deep Features [7]) with added deep features, DSST (Discriminative Scale Space Tracker [15]), MEEM (Multiple Experts Using Entropy Minimization tracker [21]), Staple (Sum of Template and Pixel-wise Leaners tracker [25]), LCT (Long-term Correlation Tracking [30]), LMCF (Large Margin Object Tracking with Circulant Feature Maps [32]), ECO-HC (Efficient Convolution Operators for Tracking based HOG and CN Features [35]), SAMF (Scale Adaptive with Multiple Features tracker [36]) and DLSSVM (Dual Linear SSVM [37]). The tracking success rate for the top 10 trackers was evaluated on all 51 videos the in the OTB-13 [33] and all 100 videos in the OTB-15 [34] benchmark datasets. These results are shown in Figure 8. As shown, MMLT performed well across the OPE, TRE, and SRE indicators. When the Staple algorithm was first proposed, it performed very well in comparison to other algorithms. Our approach achieved a 9.7% improvement on the success plots of OPE, a 7.8% improvement on the success plots of TRE, and an 8% improvement on the success plots of SRE over Staple on OTB-13, whilst also showing a similar improvement on the OTB-15 dataset relative to the Staple algorithm. We need to emphasize that our approach also ran at a significantly higher speed with 50 FPS. In addition, MMLT performed as well as DeepSRDCF with deep features. However, DeepSRDCF tracked less than 1 FPS while MMLT tracked at approximately 50 times the DeepSRDCF tracking speed. At the same time, whil using the same detection mechanism as the LCT algorithms, an average improvement of 10% on the success plots is achieved. In the meantime, our approach had an average of 1% improvement over the ECO-HC algorithm which also does not use depth features but has excellent tracking performance. Furthermore, it needs to be stressed that we compared the tracking performance with the published data where the average FPS of different trackers in the legend was the average of the tracking speed of 100 videos in the OTB-15 benchmark datasets, which was tested on our computer.

In summary, the MMLT algorithm performed well against the listed trackers, both in tracking performance and in tracking speed.

### 4.4. Attribute Based Evaluation

**Figure 9:**
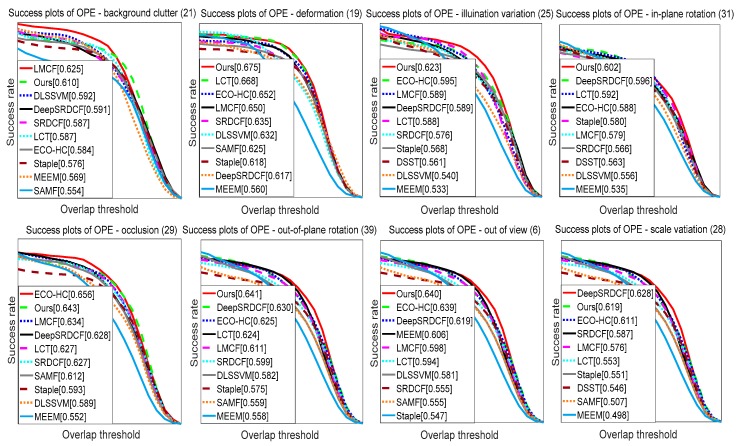
The success plots of eight challenging attributes including background clutter, deformation, illumination variation, in-plane rotation, occlusion, out of-plane rotation, out-of-view, and scale variation. The legend illustrates the ranking scores for each tracker. The proposed MMLT algorithm has five attributes ranked first, and three attributes ranked second.

The video set provided in [33] contained a selection of 11 attributes including object deformation, occlusions. These allowed for further analysis of tracker performance. Figure 9 shows the results for the eight most challenging video attributes in OTB-13 [33]. As shown, the DeepSRDCF algorithm demonstrated comprehensive performance against the existing tracking algorithms outside of trackers with depth information, i.e., in-plane rotation (59.6%), out-of-plane rotation (63.0%), and scale variation (62.8%). The MMLT algorithm achieved a success rate of 60.2%, 64.1%, and 61.9% on in-plane rotation, out-of-plane rotation, and scale variation, respectively. ECO-HC achieved the success rate of 59.5%, 65.6% and 63.9%, on illumination variation, occlusion and out of view, respectively, while MMLT achieved the success rate of 62.3%, 64.3% and 64.0%, respectively. LMCF offered a 62.5% performance on background clutter, which was matched by MMLT. LCT offered a 66.8% performance on deformation, while MMLT performed well with a success rate of 67.5%.

### 4.5. Qualitative Comparison

**Figure 10:**
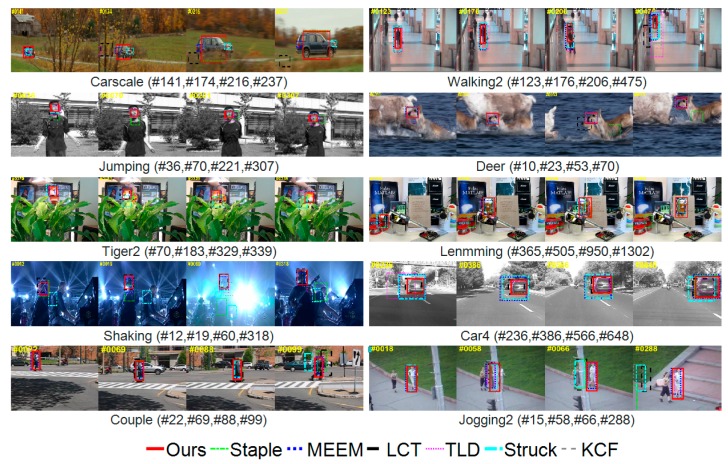
Qualitative results of our MMLT algorithm, Staple [25], MEEM [21], LCT [30], TLD [26], Struck [38], and the KCF [5] methods on ten challenging sequences. The targets in these sequences underwent heavy occlusion, motion blur, illumination variation, scale variation, and background clutter, respectively.

We compared our proposed tracking algorithm (MMLT) with six other state-of-the-art trackers, namely Staple (Sum of Template and Pixel-wise Leaners tracker [25]), MEEM (Multiple Experts Using Entropy Minimization tracker [21]), LCT (Long-term Correlation Tracking [30]), TLD (Learning and Detecting [26]), Struck (Structured Output Tracking with Kernels [38]), and KCF (Kernel Correlation Filter [5]) on ten challenging sequences (Figure 10). The Staple algorithm takes advantage of the complementary sample information by fusing the predictions of the filter model and the color model, and therefore showed good performance in handling with significant deformation and fast motion (Tiger2 and Deer). However, it drifted when the target objects underwent heavy illumination variation, occlusion, and background clutters (Shaking, Lemming, and Couple). As the color model is susceptible to changes in light and motion blur, even with the filter model as a complementary model, the tracker’s resilience was still low when the tracker encountered severe lighting changes and similar background colors; moreover, while tracking in the event of a serious occlusion, the tracking results were also prone to drift and did not re-detect targets in the case of tracking failure (Couple and Jogging-2). The MEEM tracker selected the best prediction of models collected from the past for tracking according to the minimum entropy criterion, but still did not perform well when in the presence of heavy occlusion (Walking2) or both significant scale and fast motion (Carscale). The Struck tracker did not perform well in background clutters (Shaking), fast motion (Deer), heavy occlusion, or out-of-view (Walking2, Tiger2, and Jogging-2). The KCF tracker is based on a correlation filter learned from HOG features, so drifted when the target objects underwent heavy occlusions (Lemming), and motion blur (Tiger2). In addition, the KCF tracker failed to handle background clutter (Shaking) since it is difficult to achieve robust tracking with a single feature classifier model in complex scenes. When tracking failed, the TLD tracker could re-detect the target object. However, the TLD approach did not take full advantage of the temporal movement clues and therefore did not follow targets undergoing significant deformation and fast motion (Tiger2 and Shaking) well. Moreover, the TLD method updates its detector frame-by-frame, leading to drifting. Overall, the proposed MMLT tracker performed well in estimating both the scales and positions of target objects on these challenging sequences, which can be attributed to three reasons. First, our tracker effectively combined three separate models, each dealing with different features, each complementing each other, and taking full advantage of the diversity of sample information. Therefore, in the target deformation or light, motion blur, etc., it can have a better tracking effect. Second, our confidence filter was updated only when the confidence level was high, so it could restrain the flow of the template in the detection module to a certain extent, and the object detection model incorporated into the tracker could reduce the problem of template flowing to a certain extent. Finally, we added a model detection algorithm based on the color model and object detection, which could quickly re-detect the target after it failed.

In addition, the center location errors and the average overlap rate were used to evaluate the proposed tracker. The average center location error is the average value of all the center location errors in all the video sequences, where the center location error represents the distance center between the position of predicted bounding box $R_{t}$, and the ground truth $G_{t}$ can be expressed by the criterion $CLE=\sqrt{{\left(L_{t}-L_{g,t}\right)}^{2}}$. $L_{t}$ and $L_{g,t}$ represent the location of $R_{t}$ and $G_{t}$, respectively. The average overlap rate represents the average overlap between bounding box $R_{t}$ and the ground truth $G_{t}$ in all the video sequences, and the overlap between bounding box $B_{t}$ and the ground truth $G_{t}$ can be represented by the criterion $overlap=\left|B_{t}\cap G_{t}\right|/\left|B_{t}\cup G_{t}\right|$, where ∩ represents the intersection and ∪ represents union. The average center location error and average overlap rate of the proposed algorithm on the ten sequences are shown in Table 3 and Table 4, respectively, which show the good tracking performance of our proposed tracker against the other trackers. *In addition,* the best result and the next best result are marked with red and blue in Table 3 and Table 4, respectively.

**Figure 11:**
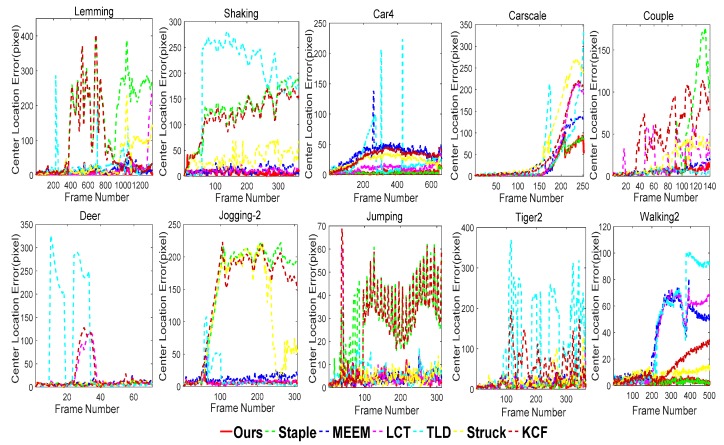
Fame-by-frame comparison of the center location errors (in pixels) on ten challenging sequences in Figure 11. Based on the experimental results, our algorithm was able to track targets accurately and stably.

Moreover, we report the central-pixel errors frame-by-frame on the ten sequences in Figure 11, which shows that our tracking algorithm performed well against the existing trackers.

### 4.6. Analysis of Merge Parameter

**Figure 12:**
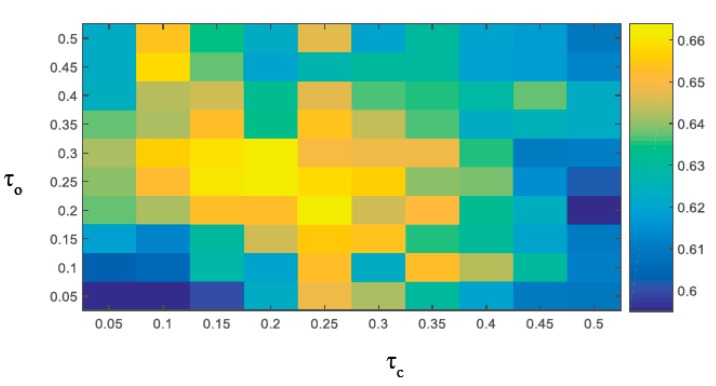
Success plots using different $\tau_{c}$ and $\tau_{o}$ values.

As shown in Figure 12, we randomly selected 25 sequences with different attributes from the OTB-13 benchmark datasets [33] and tested the effect of different merge parameters $\tau_{c}$ and $\tau_{o}$ on the TRE success rate by the cross-intersection method.

We can see in Figure 12, with the color regression model, filter regression model, and object row response model, that the tracking performance was better than the tracking performance with only the filter model and the color model, or the filter model. The performance of the three models weighting coefficients reaching the proper ratio tracker was the best. At the same time, it can also be seen in Figure 12 that the tracking success rate of the tracker was relatively high in a certain area of the optimal parameters, that is, small changes, the performance of the tracker had strong stability. Therefore, we can see that the fusion strategy based on multiple models is a good way to improve the performance of trackers. Clearly, the best performance was achieved at $\tau_{c}=0.2$ and $\tau_{o}=0.25$, which were used in the rest of our experiments.

## 5. Conclusions

In this paper, by incorporating the object model based on contour information into the translational tracking of Staple (which combines a correlation filter and color model), tracking robustness is significantly improved. Each model is responsible for tracking specific features, and then the three complementary models are combined to form a more robust tracking model. At the same time, we design a target detection method based on an object detection model based on contour features and a color model based on histogram features, which has good performance in detection efficiency and detection accuracy when compared with traditional classifier-based detection methods. In addition, we also optimize the traditional scale calculation method. The experimental results show that the proposed algorithm offers favorable improvements in performance with regard to efficiency, accuracy, and robustness.

## Figures and Tables

**Table 1:** The parameters for our experiments.

Learning Rate (Filter) $\eta_{f}$	0.01	Activation Threshold σ	0.2
Learning rate (color) $\eta_{c}$	0.04	ξ	0.7
Learning rate scale filter	0.035	χ	0.2
Color features	RGB	δ	1.3
Bins color space	32×32×32	k (object model)	1.5
Weighting coefficient (color) $\tau_{1}$	0.2	$\omega_{1}$	0.7
Weighting coefficient (object) $\tau_{2}$	0.25	$\omega_{2}$	0.45
Fix area	150^2^	HOG cell	4
$\tau_{c}$	0.2	$\tau_{o}$	0.25

**Table 2:** Tracking results for different trackers.

Trackers	Detection	Object Model	OPE		TRE		SRE		Mean FPS
			Precision	Success	Precision	Success	Precision	Success	
Staple	No	No	0.766	0.582	0.786	0.603	0.755	0.553	103
MMLT-NN	No	No	0.766	0.582	0.786	0.603	0.755	0.553	135
MMT	No	Yes	0.832	0.633	0.844	0.646	0.789	0.591	70
MMLT-N1	Yes	No	0.835	0.629	0.840	0.641	0.809	0.584	47
MMLT	Yes	Yes	0.866	0.658	0.867	0.664	0.834	0.611	50

**Table 3:** Average center location errors of the proposed method compared to other trackers (pixels).

	Ours	Staple	MEEM	LCT	TLD	Struck	KCF
Lemming	9.042	158.449	14.486	17.076	22.696	35.578	83.931
Shaking	6.568	125.060	11.179	10.357	190.684	34.825	116.893
Car4	3.013	3.071	34.829	10.388	22.160	23.231	29.131
Carscale	22.059	21.740	37.296	43.649	45.785	74.244	51.728
Couple	4.641	34.588	7.163	19.177	4.824	18.458	49.315
Deer	5.978	6.032	9.588	21.742	71.372	8.704	23.408
Jogging-2	6.156	151.059	13.192	7.467	12.386	112.456	144.996
Jumping	3.712	27.919	3.815	4.681	5.736	4.525	26.973
Tiger2	10.189	10.282	19.844	16.731	100.407	21.686	47.072
Walking2	3.115	3.178	35.236	35.706	43.908	8.793	12.797

**Table 4:** Average overlap rate of the proposed method compared to other trackers.

	Ours	Staple	MEEM	LCT	TLD	Struck	KCF
Lemming	0.748	0.232	0.650	0.701	0.500	0.481	0.347
Shaking	0.777	0.057	0.652	0.704	0.129	0.310	0.041
Car4	0.872	0.867	0.357	0.689	0.537	0.441	0.412
Carscale	0.719	0.717	0.400	0.614	0.434	0.362	0.406
Couple	0.721	0.527	0.601	0.447	0.761	0.484	0.202
Deer	0.778	0.776	0.698	0.606	0.606	0.716	0.600
Jogging-2	0.706	0.124	0.547	0.671	0.626	0.172	0.113
Jumping	0.651	0.228	0.730	0.629	0.662	0.681	0.264
Tiger2	0.689	0.687	0.536	0.611	0.232	0.546	0.357
Walking2	0.758	0.756	0.269	0.354	0.299	0.504	0.482
